# Role Of Hif2α Oxygen Sensing Pathway In Bronchial Epithelial Club Cell Proliferation

**DOI:** 10.1038/srep25357

**Published:** 2016-05-06

**Authors:** Mar Torres-Capelli, Glenn Marsboom, Qilong Oscar Yang Li, Daniel Tello, Florinda Melendez Rodriguez, Tamara Alonso, Francisco Sanchez-Madrid, Francisco García-Rio, Julio Ancochea, Julián Aragonés

**Affiliations:** 1 Research Unit, Santa Cristina Hospital, Research Institute Princesa (IP), Autonomous University of Madrid, Madrid 28009, Spain; 2 Department of Pharmacology, University of Illinois College of Medicine, Chicago, IL 60612, USA; 3 Pulmonology Department, La Princesa Hospital, Research Institute Princesa (IP) Madrid, Spain; 4 Pulmonology Department, La Paz Hospital, IdiPAZ, Madrid, Spain; Autonomous University of Madrid, Madrid, Spain CIBER -CIBERES, Madrid, Spain

## Abstract

Oxygen-sensing pathways executed by the hypoxia-inducible factors (HIFs) induce a cellular adaptive program when oxygen supply becomes limited. However, the role of the HIF oxygen-sensing pathway in the airway response to hypoxic stress in adulthood remains poorly understood. Here we found that *in vivo* exposure to hypoxia led to a profound increase in bronchial epithelial cell proliferation mainly confined to Club (Clara) cells. Interestingly, this response was executed by hypoxia-inducible factor 2α (HIF2α), which controls the expression of FoxM1, a recognized proliferative factor of Club cells. Furthermore, HIF2α induced the expression of the resistin-like molecules α and β (RELMα and β), previously considered bronchial epithelial growth factors. Importantly, despite the central role of HIF2α, this proliferative response was not initiated by *in vivo Vhl* gene inactivation or pharmacological inhibition of prolyl hydroxylase oxygen sensors, indicating the molecular complexity of this response and the possible participation of other oxygen-sensing pathways. Club cells are principally involved in protection and maintenance of bronchial epithelium. Thus, our findings identify a novel molecular link between HIF2α and Club cell biology that can be regarded as a new HIF2α-dependent mechanism involved in bronchial epithelium adaptation to oxygen fluctuations.

Cellular adaptation to oxygen fluctuations is driven by oxygen-sensing pathways such as those mediated by the hypoxia-inducible factors (HIF1, HIF2 and HIF3), central regulators of the transcriptional response to hypoxia. Under normoxic conditions, prolyl hydroxylases (PHD− 1,− 2 and − 3) use oxygen to hydroxylate critical proline residues in HIFα subunits, which ultimately leads to their recognition by the von Hippel-Lindau (*Vhl*) protein and subsequent degradation by the proteasome^1^,^2^,^3^,^4^. Additionally, the asparagine hydroxylase, factor inhibiting HIF (FIH), hydroxylates the C-terminal domain of HIFα subunits, which prevents the binding of the transcriptional coactivator p300 and therefore HIF transactivation potential^5^,^6^. PHD and FIH activities are reduced in hypoxia and HIFα subunits accumulate and activate HIF-dependent transcriptional programs. Accordingly, pharmacological inhibition of PHD and FIH activities or inactivation of *Vhl*, *Phd* or *Fih* genes, results in constitutive normoxic stabilization or activity of HIF transcription factors^5^,^6^,^7^,^8^.

Erythropoiesis and angiogenesis are archetypal adaptive physiological responses to hypoxia, mediated largely by the ability of HIFs to govern the expression of erythropoietin (EPO)^9^ and vascular endothelial growth factor (VEGF)^10^. Inhibition of cell-autonomous replication is an additional classic response to hypoxia; indeed, hypoxia attenuates cell proliferation in several different cell types^11^. Several mechanisms have been proposed to promote this antiproliferative action including HIF1α -dependent inhibition of c-MYC activity^12^,^13^ or repression of DNA replication by a HIF1α -dependent non-transcriptional mechanism that ultimately compromises the activity of MCM DNA helicase^14^. Furthermore, inhibition of PHD1 reduces cyclinD1 expression in breast cancer cells independently of HIF^15^, and promotes cell cycle arrest through regulation of the centrosomal scaffold protein CEP192^16^. Reduced oxygen environments and subsequent activation of the HIF pathway are not, however, invariably associated with attenuation of cell cycle and proliferation. For example, hypoxia promotes the proliferation of neural stem cells (NSC)^17^,^18^. Indeed, HIF1α plays a significant role in regulating cell-autonomous NSC proliferation *via* modulation of the expression of the β -catenin effectors LEF-1 and TCF-1, and enhances Wnt/ β -catenin proliferative signalling^19^. Additionally, in *Vhl*-deficient renal carcinoma cells, HIF2α promotes cell-autonomous proliferation and tumour growth^12^,^20^,^21^,^22^.

The role of the HIF oxygen sensing pathways in lung biology has been mainly associated with pulmonary hypertension as a consequence of HIF-dependent pulmonary artery smooth muscle cell (PASMC) proliferation and vascular remodelling in response to chronic hypoxia (several weeks)^23^,^24^. However, the role of HIF oxygen-sensing pathways in the response of lung conducting airways to hypoxic stress remains largely unknown. Nevertheless, a recent study has shown that lower HIF2α levels are associated with multiple severity phenotypes of chronic obstructive pulmonary disease (COPD) in humans, and emphysema severity-associated genes in mice^25^. Along this line, it was recently found that loss of the HIF2α isoform in airway cells leads to enhanced airway inflammation upon lung injury^26^. In the present study, we have identified Club (Clara) bronchial epithelial cells, previously involved in the maintenance and protection of bronchial epithelium, as a novel cell type that displays a remarkable proliferative potential upon *in vivo* exposure to hypoxia. This response requires HIF2α , which leads to the upregulation of FoxM1 as well as the established bronchial epithelial cell growth factors RELMα and RELMβ . However, HIF2α activation by pharmacological inhibition of PHD activity is not sufficient to induce Club cell proliferation. Our data therefore demonstrate that proliferation of bronchial Club cells is part of the biological responses initiated by the HIF2α oxygen-sensing pathway in lung tissue.

## Results

### Hypoxia induces proliferation of bronchial epithelial cells

**Figure 1:**
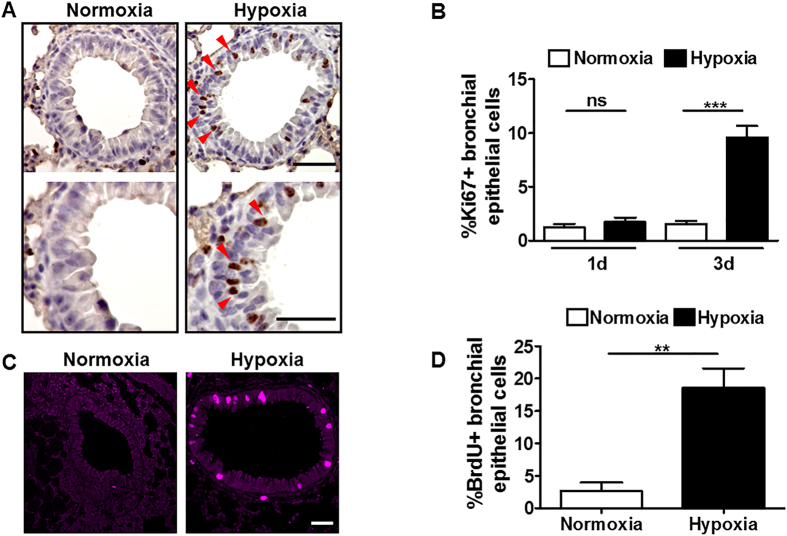
Hypoxia induces proliferation of bronchial epithelial cells. (**A**) Ki67 immunostaining in lung sections of control mice exposed to normoxia or hypoxia (10% O_2_) for 3 days. Red arrowheads indicate Ki67^positive^ bronchial epithelial cells. Images of the lower panels are higher magnification of those in the upper panels. (**B**) Quantification of Ki67^positive^ bronchial epithelial cells in lung of control mice exposed to normoxia (n =  15) or hypoxia 10% O_2_ (n =  15) for 1 day as well as to normoxia (n =  12) or hypoxia 10% O_2_ (n =  16) for 3 days. (**C**) BrdU immunofluorescence in the lung of control mice exposed to normoxia or hypoxia (10% O_2_) for 4 days. (**D**) Quantification of BrdU^positive^ bronchial epithelial cells in lung of control mice exposed to normoxia (n =  5) or hypoxia 10% O_2_ (n =  5) for 4 days. For (**B**,**D**), n indicates the number of bronchial epithelia examined; the mean is shown, and error bars represent SEM. Statistical significance was assessed by the two-tailed Student’s t test: **p <  0.01; ***p <  0.001; ns (not significant). Scale bars: (**A**) Upper panel, 50 μ m, lower panel, 20 μ m; (**C**), 50 μ m.

Lung is the first-line contact organ with oxygen but the responses of lung conducting airways to hypoxic stress *in vivo* remains poorly understood. It is recognized that chronic hypoxia promotes pulmonary vascular smooth muscle cell proliferation, leading to vascular remodelling and pulmonary hypertension^27^. Here, we studied the pulmonary responses to oxygen fluctuations in mice subjected to hypoxia (10% O_2_), but at shorter time points (1-4 days). We first studied cell proliferation in lungs from mice exposed to normoxia or hypoxia by staining lung sections with the cell cycle-associated protein Ki67, a marker of cell proliferation. Surprisingly, immunohistochemistry analysis showed a clear increase in the number of Ki67^positive^ cells in lung conducting airways after exposure of mice to hypoxia for 3 days, and this was particularly manifested in the bronchial epithelium (Fig. 1A). Quantification of Ki67 staining revealed that after 3 days of hypoxia exposure, approximately 10-15% of bronchial epithelial cells were Ki67^positive^ (Fig. 1B). Moreover, this response required more than 1 day to be mounted because no increase in the number of Ki67^positive^ bronchial epithelial cells after 1 day of hypoxia exposure was detected (Fig. 1B). To confirm that hypoxic bronchial epithelial cells enter into a proliferative state, mice were exposed to hypoxia or normoxia and injected intraperitoneally with the DNA synthesis marker BrdU. Analysis of BrdU staining in lung sections from hypoxic animals showed the appearance of BrdU^positive^ cells in hypoxic lung conducting airways that was evident in bronchial epithelial cells (Fig. 1C). Quantification of BrdU^positive^ bronchial epithelial cells revealed a significant increase in their percentage in mice exposed to hypoxia (Fig. 1D). Collectively, these data show that low oxygen tension *in vivo* initiates a rapid proliferative response in lung conducting airway cells, which is particularly evident in bronchial epithelial cells.

### Hypoxia induces proliferation of bronchial Club epithelial cells

**Figure 2:**
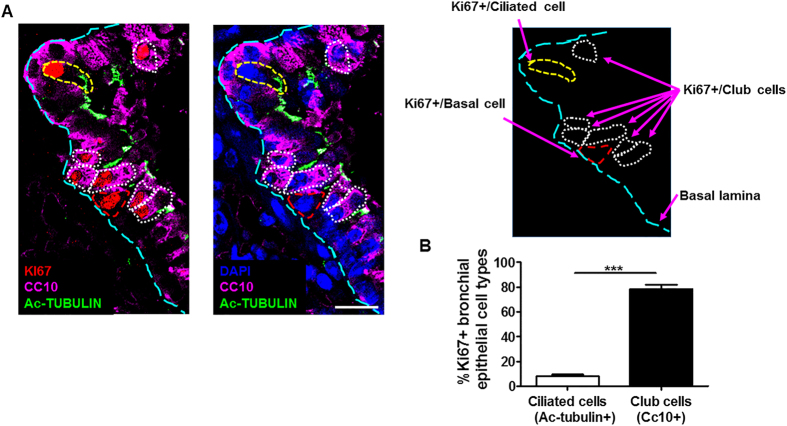
Hypoxia induces proliferation of bronchial Club epithelial cells. (**A**) Immunofluorescence of Club (CC10, purple), ciliated (acetyl-tubulin, green) and proliferative cells (Ki67, red) in lung sections of control mice exposed to hypoxia (10% O_2_) for 3 days (left panel). Consecutive lung section stained with CC10 (purple) and acetyl-tubulin (green) antibodies together with DAPI (blue) to localize nuclei of bronchial epithelial cells (middle panel). In the right panel, a representation indicating the meaning of the dotted lines is shown. (**B**) Quantification of Ki67^positive^ ciliated or Club cells relative to total Ki67^positive^ bronchial epithelial cells (n =  20). n indicates the number of bronchial epithelia examined. Values are expressed as mean ±  SEM and unpaired Student’s t test was used for statistical analysis: ***p <  0.001. Scale bar: 10 μ m.

Club cells and ciliated cells are two essential cell types of the bronchial epithelium, and they can be detected using specific markers. Accordingly, Club cells are detected with a specific antibody against CC10, a secretory product also known as secretoglobin family 1A member 1 (SCGB1A1)^28^, whereas ciliated cells are detected with a specific antibody against acetyl-tubulin, a protein expressed in primary and motile cilia within the cytoplasm^28^. We used these markers to determine whether bronchial epithelial cell proliferation in hypoxic lungs was preferentially due to any of these bronchial epithelium cell types. Thus, lung sections of mice subjected to hypoxia for 3 days were co-stained with antibodies against Ki67, CC10 and acetyl-tubulin to assess proliferation in Club or ciliated cells, respectively. Immunohistochemistry analysis revealed that the majority of Ki67^positive^ signals were confined to CC10^positive^ cells (Fig. 2A). By contrast, the number of cells with double-positive Ki67/acetyl-tubulin staining was less abundant (Fig. 2A). Quantification of the signals showed that 78.31 ±  3.54% of Ki67^positive^ cells were CC10^positive^ cells whereas only 8.11 ±  1.36% were acetyl-tubulin^positive^ cells (Fig. 2B). This analysis also detected the presence of some Ki67^positive^ cells located in the basal lamina lacking the expression of CC10 and acetyl-tubulin (13.58 ±  2.63%), possibly reflecting some proliferation in bronchial epithelium basal stem cells. Collectively, these data clearly indicate that low oxygen tension induces bronchial epithelium proliferation *in vivo*, which is mainly confined to Club cells.

### Hypoxia-induced bronchial epithelial cell proliferation requires HIF2α

**Figure 3:**
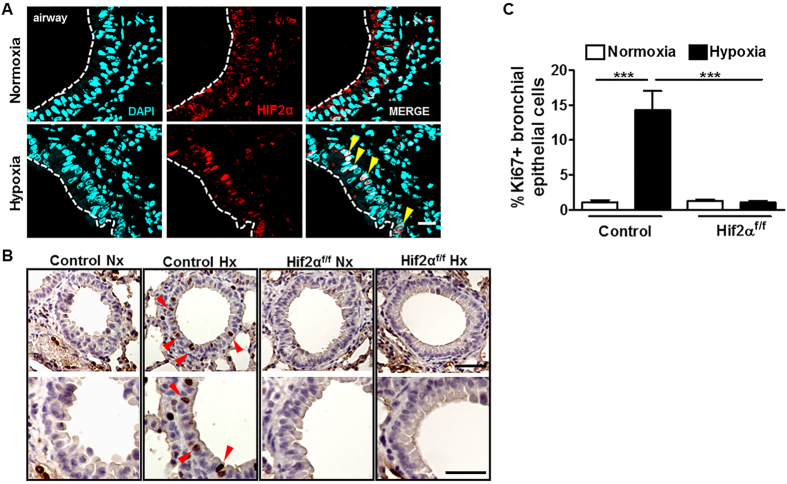
Hypoxia-induced bronchial epithelial cell proliferation requires HIF2α. (**A**) HIF2α immunostaining in lung sections of control mice exposed to normoxia or hypoxia (10% O_2_) for 3 days. DAPI nuclear counterstaining shows colocalization of HIF2α and nuclei signal of hypoxic bronchial epithelial cells (yellow arrowheads). (**B**) Immunostaining for Ki67 in lung sections of control or HIF2α -deficient mice (HIF2α ^floxed^-Ubc-Cre-ER^T2^) exposed to normoxia or hypoxia (10% O_2_) for 3 days. Red arrowheads indicate Ki67 positive bronchial epithelial cells. (**C**) Quantification of Ki67 positive bronchial epithelial cells in control exposed to normoxia (n =  10) or hypoxia 10% O_2_ (n =  11) for 3 days or HIF2α -deficient mice exposed to normoxia (n =  18) or hypoxia 10% O_2_ (n =  31) for 3 days. *n* indicates the number of bronchial epithelia analysed. Values are expressed as mean ±  SEM and statistical significance was assessed by the One-way ANOVA Tukey’spost test. ***p <  0.001. Scale bars: (**A**), 10 μ m; (**B**), upper panel, 50 μ m, lower panel, 20 μ m.

Club cells and their secretory protein CC10 are mainly orientated towards the protection of the respiratory tract, and serum levels of CC10 are reduced in individuals with COPD^29^,^30^. Furthermore, recent studies have shown that lower levels of HIF2α are associated with COPD severity in human and mice^25^. Considering that rodent lung tissue expresses high levels of HIF2α RNA^31^, we hypothesized that the HIF2α oxygen-sensing pathway could have a direct role in hypoxia-driven bronchial epithelial cell proliferation. We performed an immunofluorescence analysis to assess whether HIF2α was expressed in hypoxic bronchial epithelium. Our analysis localized a robust HIF2α signal in the nuclei of bronchial epithelial cells of hypoxic lungs, while this nuclear signal was barely present in normoxic lungs (Fig. 3A). Therefore, these data show that both hypoxia-driven bronchial epithelium proliferation as well as the appearance of nuclear HIF2α is observed in bronchial epithelial cells. To investigate the involvement of HIF2α in bronchial epithelial cell proliferation, we performed Ki67 staining in lung sections of control mice and HIF2α ^floxed^-Ubc-Cre-ER^T2^ mice (HIF2α ^f/f^ deficient mice), in which the HIF2α locus can be acutely inactivated in adult mice^22^. Indeed, in this study, HIF2α gene expression was reduced by 90.6 ±  1.9% in HIF2α ^floxed^-Ubc-Cre-ER^T2^ relative to control mice. Consistent with earlier results (Fig. 1B), the number of Ki67^positive^ cells increased significantly in response to hypoxia in control mice, but this response was completely abolished in HIF2α ^f/f^ deficient mice (Fig. 3B,C). These data show that HIF2α is required for hypoxia-induced bronchial epithelium proliferation.

**Figure 4:**
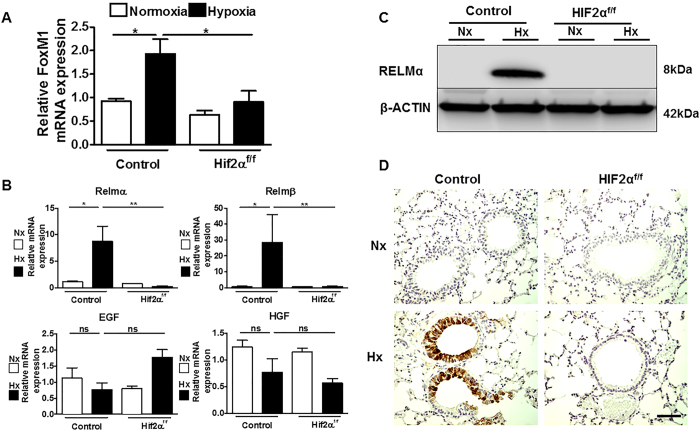
Hypoxia induces FoxM1 and RELMα in hypoxic lungs through HIF2α. (**A**) Relative FoxM1 mRNA levels (normalized to those of Hprt) in the lungs of control mice exposed to normoxia (n =  4) or hypoxia 10% O_2_ (n =  6) for 3 days or HIF2α -deficient mice exposed to normoxia (n =  3) or hypoxia 10% O_2_ (n =  4) for 3 days. (**B**) Relative RELM-α , RELM-β , EGF and HGF mRNA levels (normalized to that of Hprt) in the lungs of control mice exposed to normoxia (n =  3) or hypoxia 10% O_2_ (n =  4) for 3 days or HIF2α -deficient mice exposed to normoxia (n =  3) or hypoxia 10% O_2_ (n =  4) for 3 days. (**C**) Western blot analysis of RELM-α and β –actin protein levels in the lungs of control or HIF2α -deficient mice exposed to normoxia or hypoxia (10% O_2_) for 3 days. A representative western blot is shown. (**D**) RELM-α immunohistochemistry in lung sections of control or HIF2α -deficient mice exposed to normoxia or hypoxia (10% O_2_) for 3 days. For (**A**,**B**) panels values are expressed as mean ±  SEM and *n* is the number of the animals analysed. Error bars show 95% confidence interval based on duplicated samples. One-way ANOVA Tukey’spost test was used for statistical analysis. *p <  0.05; **p <  0.01; ns (not significant). For (**D**) panel, scale bar: 50 μ m.

Regarding potential HIF2α downstream events that could be involved in this proliferative response, we first considered previous *in vivo* studies showing that the FoxM1 transcription factor is critical for proliferation and differentiation of Club cells^32^,^33^. RNA analysis showed that FoxM1 RNA levels were induced by hypoxia in control mice, but this response was impaired in HIF2α ^f/f^ deficient mice (Fig. 4A). Moreover, we also considered previous *in vitro* studies implicating several soluble factors in bronchial epithelium proliferation, such as resistin-like molecules (RELM), epithelial growth factor (EGF) and hepatic growth factor (HGF)^34^,^35^,^36^,^37^. RNA analysis showed that the expression levels of RELMα and RELMβ in control mice were significantly higher in hypoxia than in normoxia, whereas EGF and HGF were unaffected by oxygen tension (Fig. 4B). Western blotting revealed that whereas RELMα protein was undetectable in normoxic lungs, it was strongly induced after 3 days of hypoxia (Fig. 4C). Hypoxia-induced expression of RELMα RNA and protein was completely abolished in HIF2α ^f/f^ deficient mice, showing that its expression is HIF2α dependent in hypoxic lungs (Fig. 4B,C). Immunohistochemistry analysis further showed that HIF2α -dependent RELMα expression was largely localized to bronchial epithelium (Fig. 4D). Overall, these data show that HIF2α activity is required for epithelial cell proliferation in hypoxic bronchial epithelium, and controls key regulators of Club cell proliferation, such as FoxM1, as well as the previously established bronchial epithelium mitogen RELMα .

### *Vhl* gene inactivation or pharmacological inhibition of PHD oxygen sensors is not sufficient to induce bronchial epithelial cell proliferation

**Figure 5:**
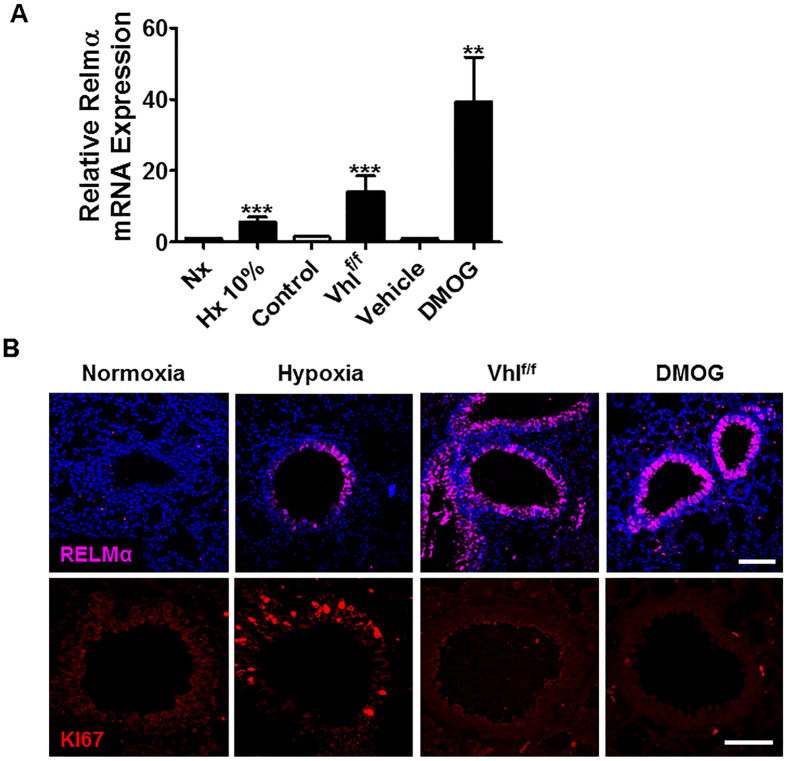
*Vhl* gene inactivation or DMOG treatment does not induce bronchial epithelium proliferation. (**A**) Relative RELMα mRNA levels (normalized to those of Hprt) in mice exposed to normoxia (n =  3) or hypoxia 10% O_2_ (n =  6) for 3 days, *Vhl* deficient mice (n =  9) and their corresponding control mice (n =  9) as well as DMOG treated mice (n =  3) or vehicle-treated mice (n =  2). (**B**) Immunofluorescence of RELM-α (purple) or Ki67 (red) in lung of control mice, hypoxia-exposed mice (10% O_2_ for 3 days), *Vhl*-deficient mice and DMOG-treated mice. Data shown are means ±  SEM and *n* is the number of mice examined. Error bars show 95% confidence interval based on duplicated samples. The differences between groups with similar variances were analysed with a two-tailed Student´s t-test:**p <  0.01; ***p <  0.001. Scale bars: (**B**), upper and lower panel, 50 μ m.

Since Club cells are associated with respiratory tract protection, we next questioned whether constitutive HIF2α activation in normoxia was sufficient to promote Club cell proliferation. To do this, we used adult *Vhl*^floxed^-Ubc-Cre-ER^T2^ mice in which the expression of *Vhl*, a central repressor of HIF activity, can be acutely inactivated and leads to an elevated expression of HIFα protein subunits in lung^22^. To corroborate that *Vhl* inactivation also leads to HIF2α activity in lung, the expression of RELMα was evaluated as a HIF2α -dependent gene in *Vhl*^floxed^-Ubc-Cre-ER^T2^ mice. RELMα gene expression in lung was significantly increased in these mice as in hypoxia-exposed mice (Fig. 5A). Furthermore, consistent with its localization in control mice, immunohistochemistry analysis revealed that RELMα protein expression was confined largely to the bronchial epithelium in *Vhl*-deficient mice (Fig. 5B). Therefore, *Vhl*^floxed^-Ubc-Cre-ER^T2^ mice accurately mimic the phenotype of hypoxia-induced HIF2α activity measured as RELMα expression in the bronchial epithelium. Nevertheless, in contrast to hypoxia-treated mice, the number of Ki67^positive^ cells in the bronchial epithelium was not elevated in *Vhl*-deficient mice (Fig. 5B). These data suggest that HIF2α activation in *Vhl*-deficient mice is not sufficient to promote bronchial epithelium proliferation. Therefore, as a second approach to induce HIF2α activity in lung, we treated mice with dimethyloxalylglycine (DMOG), a competitive inhibitor of PHD oxygen sensors. The expression of RELMα was also evaluated as a readout of HIF2α activity in DMOG-treated mice. Pulmonary RELMα expression was elevated both at the RNA level (Fig. 5A) and at the protein level, and protein expression was mainly localized at the bronchial epithelium (Fig. 5B). Thus, DMOG treatment also mimicked the effect of hypoxia on HIF2α activity and RELMα expression in the bronchial epithelium. The efficacy of DMOG treatment was also evaluated by its ability to induce Epo gene expression in the kidneys of these DMOG-treated mice (Supplemental Fig. 1). Nevertheless, DMOG treatment failed to increase the number of Ki67^positive^ cells in the bronchial epithelium. Collectively, these results indicate that although HIF2α is required to promote Club cell proliferation, it is not sufficient.

In conclusion, our data identify a novel molecular response initiated by HIF2α , which promotes the proliferation of Club cells, which are known to be involved in bronchial epithelial maintenance and protection. Importantly, our findings also reveal that HIF2α activation is required but it is not sufficient to mount this proliferative response, underscoring the molecular complexity of this oxygen-dependent pulmonary proliferative response.

## Discussion

Pulmonary HIF oxygen sensing pathways are associated with vessel remodelling and pulmonary hypertension in response to chronic hypoxia^23^,^24^,^38^. However, their role in the response of lung conducting airways to hypoxic stress in adulthood is largely unknown. Here we show that the HIF2α isoform promotes bronchial epithelial cell proliferation soon after hypoxia exposure and is mainly confined to Club cells. A recent study has shown that lower HIF2α levels are associated with multiple COPD severity phenotypes in human and emphysema severity-associated genes in mouse^25^. This, together with the contribution of Club cells to the long-term maintenance and repair of lung airway epithelium, by proliferating in response to epithelial damage to reconstitute injured airways^39^, suggest a possible reparative role for Club cells in lung disease involving the HIF2α isoform. Moreover, oxidative damage of the lung has emerged as a novel primary mechanism leading to lung damage in COPD and hypoxic lung injury^40^. Indeed, reactive oxygen species (ROS) from cigarette smoke are proposed as a primary cause of an amplified inflammation leading to apoptosis and irreversible lung damage^40^,^41^. It is also important to stress that the HIF2α pathway can also counteract oxidative damage in different biological settings^7^,^42^. In this context, it is important to consider that Club cells and their secretory protein CC10 have been shown to have lung protective properties^43^,^44^,^45^. Indeed, prolonged exposure to cigarette smoke reduces Club cell activity and CC10 levels in bronchoalveolar lavage fluids of healthy smokers^43^ and reduced CC10 levels are associated with oxidative stress and inflammation that results in reduced pulmonary function and COPD development^44^. Furthermore, serum levels of CC10 are reduced in individuals with COPD and are considered a biomarker of this lung disease^29^. Therefore, it is conceivable that HIF2α -driven Club cell proliferation could also act as a cytoprotective axis to protect airway cells against oxidative stress and inflammation. Finally, recent studies also suggest the ability of HIF2α to initiate anti-inflammatory pathways. Indeed, HIF2α gene inactivation exacerbates pulmonary eosinophilic inflammation upon cobalt chloride-induced lung injury^26^. Therefore, it could be considered firstly that potentiation of HIF2α could attenuate lung dysfunction in COPD by counteracting associated oxidative stress, cell damage and inflammation, and secondly a possible contribution of HIF2α -induced Club cell proliferation.

Regarding the molecular mechanisms underlying hypoxia-mediated changes in cell proliferation, we mainly focused on the role of HIF2α since hypoxia-induced bronchial epithelial cell proliferation is completely impaired upon HIF2α gene inactivation, and also that HIF2α expression and activity are induced in bronchial epithelial cells (Fig. 3A–C). This fits well with the finding that HIF2α is highly abundant in the lung^23^,^31^, and particularly so in airway cells as shown in this study. Previous studies have demonstrated a role for HIF1α in pulmonary hypertension induced upon chronic exposure to the same hypoxic conditions (10% O_2_) as used in our study^24^,^46^. We cannot rule out the possibility that HIF1α is also active in bronchial epithelial cells in our hypoxia-exposed mice. In this line, it is conceivable that both isoforms are not induced to the same extent at a given oxygen tension (i.e. 10% O_2_ in our study). Consistent with this notion, a previous study showed that pulmonary expression of HIF1α in ventilated lungs requires more severe hypoxia than 10% O_2_^46^, which could reflect perhaps different sensitivities of each isoform to the levels of pulmonary hypoxia. In this regard, other studies have also shown that HIF2α can be induced at more modest hypoxic conditions than HIF1α 47. It is conceivable that HIF2α stabilization in hypoxic lungs requires milder hypoxic oxygen tensions than HIF1α , and perhaps HIF2α activity is more prominent than HIF1α at the hypoxic conditions required to promote bronchial epithelial cell proliferation. It should also be considered that HIF1α acts as a repressor of cell proliferation in some biological settings^11^,^12^,^13^,^14^, which could potentially compromise HIF2α -dependent bronchial epithelial cell proliferation. Considering that the lung of *Vhl* deficient mice upregulates both HIF1α and HIF2α , as shown by western blotting^22^, it is plausible that a profound activation of HIF1α upon *Vhl* gene inactivation might explain, to some extent, the absence of proliferative bronchial epithelial cells in *Vhl*-deficient mice. Nevertheless, these recognized antiproliferative mechanisms executed by HIF1α are unlikely to be prevalent in hypoxic bronchial epithelium since the contrary response occurs, illustrated by the increased number of proliferative bronchial epithelial cells.

Regarding the potential molecular mechanisms executed by HIF2α to promote bronchial epithelial cell proliferation, we found a significant induction of HIF2α -dependent expression of FoxM1. *In vitro* studies have implicated this factor in hypoxia-induced PASMC proliferation^48^, but recent *in vivo* studies have shown that it is essential to confer Club cell proliferation and differentiation during embryonic development^32^,^33^. These *in vivo* studies as well as the concomitant appearance of Ki67^positive^ and BrdU^positive^ Club cells and elevated FoxM1 expression suggest its participation in HIF2α -dependent Club cell proliferation. Previous studies have shown oxygen-dependent regulation of RELMα 49,50. RELMα expression is driven by HIF2α and is localized in bronchial epithelium in hypoxic mice. Along this line, RELMβ , the only member of the resistin-like molecules expressed in human, can act as an airway-remodelling mediator promoting epithelial cell proliferation^34^, which might also contribute to HIF2α -dependent bronchial epithelium proliferation. A similar mechanism has been proposed to explain NSC proliferation through HIFs, but involving the release of extracellular HIF-dependent factors such as EPO and VEGF^51^,^52^. However, HIF2α can also act as a pro-proliferative factor in other scenarios, such as *Vhl*-deficient renal cell carcinoma or some stem cell niches, through a number cell-autonomous mechanisms including activation of genes involved in the cell cycle in addition to mTORC1 activation^12^,^21^,^22^,^53^,^54^.

Our findings, however, also indicate that mechanistically, Club cell proliferation is complex since constitutive activation of the PHD/HIF axis in *Vhl*-deficient mice as well as in DMOG-treated mice failed to induce Club cell proliferation. In particular, *Vhl* gene inactivation is sufficient to elevate HIF2α -dependent RELMα expression to a higher level that in hypoxia-exposed mice (Fig. 5A,B), although presumably FIH is still active. Along this line, a previous study has shown that hydroxylation of HIF2α by FIH is less efficient than hydroxylation of HIF1α 55. Therefore, it is possible that full activity of HIF2α is more easily achieved than HIF1α upon *Vhl* inactivation. These data reflect the fact that HIF2α activity is effectively induced in bronchial epithelium of *Vhl*-deficient mice, suggesting that failure to increase the number of Ki67-positive cells in the bronchial epithelium in *Vhl*-deficient mice cannot be simply attributed to inefficient HIF2α activation in these mice. However, it is still possible that the presumed persistent activity of FIH on HIF2α upon *Vhl* gene inactivation might preclude a full HIF2α activation required to promote bronchial epithelial cell proliferation. Indeed, some HIF dependent genes are particularly controlled by FIH^8^,^56^ and they could remain underexpressed in *Vhl*-deficient cells. In addition to these considerations, *Vhl* deficiency as well as DMOG treatment might affect other *Vhl* targets different from HIF factors, in addition to inhibition of other 2-oxoglutarate-dependent dioxygenases that could ultimately impede HIF2α -dependent bronchial epithelial proliferation. But our data also point to HIF-independent mechanisms. In this regard, recent studies show that carotid body (CB) sustentacular cell proliferation in hypoxic conditions requires HIF2α  57, but also a HIF-independent oxygen-sensing mechanism initiated in the adjacent neuron-like glomus cells type I cells^58^. Indeed, the appearance of BrdU-positive cells in the CB of hypoxic wild-type mice is severely impaired in hypoxic HIF2α -deficient mice^57^. However, *in vivo* administration of DMOG failed to promote such proliferative responses^58^. Similarly, a recent study has shown that glomus cells rapidly release endothelin-1 that acts on endothelin receptor-expressing type II cells to instruct their growth^58^,^59^. Therefore, similar to CB sustentacular cells, Club cell proliferation might involve HIF independent pathways, which could explain why constitutive pulmonary HIF2α activation is not sufficient to promote a full bronchial epithelium proliferative response.

In conclusion, our data show that *in vivo* exposure to hypoxia stress leads to a remarkable proliferation of bronchial epithelium mainly confined in Club cells, which can be regarded as a compensatory and adaptive response because Club cells have been previously associated with airway cell regeneration and protective properties in pulmonary disease settings such as COPD. We also highlight the molecular complexity of this response involving the participation of HIF2α -dependent mechanisms, such as FoxM1 and RELMs, as well as HIF-independent mechanisms. Therefore, our findings establish a novel molecular link between hypoxic stress, HIF2α and Club bronchial epithelial cells, which are central in lung conducting airway biology.

## Material and Methods

### Ethics statement

All experimental procedures were approved by the Research Ethics Committee at the UAM and were carried out under the supervision of the Head of Animal Welfare and Health at the UAM in accordance with Spanish and European guidelines (RD 53/2013, 1 February 2013, and 2010/63/UE European Council Directive).

### Animal models

*Vhl* floxed-Ubc-Cre-ERT2 mice and corresponding controls were generated as described^60^ using C;129S-*Vhl*htm1Jae/J mice (The Jackson Laboratory, stock no. 4081) and B6.Cg-Tg(UBC-Cre/ERT2)1Ejb/J mice (The Jackson Laboratory, stock no. 008085), which ubiquitously express a tamoxifen-inducible Cre recombinase (Cre-ERT2). Epas1tm1Mcs/J mice (The Jackson Laboratory, stock no. 008407) and B6.Cg-Tg(UBC-Cre/ERT2)1Ejb/J mice were used to generate HIF2α floxed-UBC-Cre-ERT2 mice. Epas1tm1Mcs/J mice harbour two loxP sites flanking exon 2 of the murine HIF2α locus. For gene inactivation, mice were fed *ad libitum* with Teckland CRD TAM400/CreER tamoxifen pellets (Harlan Teklad) for 10-15 days and were later returned to standard mouse chow diet (Safe^®^ , Augy, France). HIF2α ^floxed^ mice or *Vhl*^floxed^ lacking UBC-Cre-ER^T2^ were used as control mice.

### *In vivo* hypoxic treatment and drug administration

#### Hypoxic conditions

To induce hypoxia *in vivo*, mice were placed in an airtight chamber with inflow and outflow valves, which was infused with a mixture of 10% O_2_, 90% N_2_ (S.E. Carburos Metalicos S.A) during 3–4 days.

#### DMOG treatment

DMOG (Enzo Lifesciences) was prepared in PBS. DMOG was administered once daily for 3 days at a dose of 16 mg DMOG per mouse by intraperitoneal injection (i.p.). Control mice were injected in the same conditions with PBS. Mice were sacrificed 24 hours post injection after the last day.

#### BrdU Injection

Mice were exposed to normoxia or 10% hypoxia for four days. On the third and fourth day, mice were treated with 3 mg BrdU i.p. (Sigma). Mice were sacrificed 4 h after the second BrdU injection.

### RNA extraction and qRT-PCR analysis and primers

Pulverized mouse lungs were processed as previously and snap-frozen in liquid nitrogen^22^. Lung tissue was homogenized in Trizol (Invitrogen) with two freeze/thaw cycles and total RNA was isolated using the RNeasy RNA Extraction Kit (Qiagen). A cDNA template was prepared by reverse transcription of RNA (1 μ g) using Improm-II reverse transcriptase (Promega) and gene expression was then assessed using a Power SYBR Green PCR Master Mix kit (Applied Biosystems). Amplification data were analysed using StepOne Software version 2.0 (Applied Biosystems). The following mouse primer sets were used: *Hprt1* (Fw 5′-GTTAAGCAGTACAGCCCCAAA-3′; Rv 5′-AGGGCATATCCAACAACAAACTT-3′), *Hif2α* (Fw 5′-ATGCCCTGGATTCGGAGA-3′; Rv 5′-GATACCACCTGCCCCTTGGT-3′), *Relmα* (Fw 5′-AACTTCTTGCCAATCCAGCTAACTA-3′; Rv 5′-AGCCACAAGCACACCCAGTAG-3′); *Relmβ* (Fw 5′-CAAAGGATCAAGGAA GCTCTCAGT-3′; Rv 5′-AGCCATAGCCACAAGCACATC-3′); *FoxM1* (Fw 5′-CACTTGGATTGAGGACCACTT-3′; Rv 5′-GTCGTTTCTGCTGTGATTCC-3′); *Hgf* (Fw 5′-CTGACACCCCTTGGGAGTATTG-3′; Rv 5′-GGTATTGCTGGT TCCCCTGTAA-3′), *Egf* (Fw 5′-CTTCAGGACCACAGCCACTTTTA-3′; Rv 5′-ATCCAAGGCAAAAACCATTCC-3′) and *Epo* (Fw 5′-TCATCTGCGACAGTCGAGTTCT-3′; Rv 5′-TTTTCACTCAGTCTGGGACCTTCT-3′).

### Western blotting

Pulverized mouse lungs were homogenized in RIPA buffer (50 mM Tris HCl, pH 7.4, 1% Triton X-100, 0.2% SDS, 1 mM EDTA) supplemented with an EDTA-free protease inhibitor (Roche) and protein extracts were quantified. Western blotting was performed using 15% SDS-polyacrylamide gels and membranes were probed with antibodies raised against β -actin (Santa Cruz, sc1616, 1:1000) and RELMα (Abcam, ab39626, 1:1000). Blots were incubated with horseradish peroxidase-linked secondary antibodies and immunoreactivity was detected by enhanced chemiluminescence (SuperSignal West Femto Maximum Sensitivity Substrate, Thermo Scientific) and visualized with a digital luminescent image analyzer (Image Quant LAS4000 Mini; GE).

### Histological Analysis

Lung tissue was embedded in paraffin after overnight fixation in 4% paraformaldehyde. Microwave-induced antigen retrieval (15′ at 240 W) was performed in 0.01 M sodium citrate (pH 6). For immunohistochemistry analysis, endogenous peroxidase was blocked with 3% H2O2 in methanol. Antibody binding was visualized using a LSAB +  Peroxidase Kit with 3,3′ -diaminobenzidine as the chromogen (Dako). Sections were dehydrated and mounted with Eukitt mounting medium (Sigma-Aldrich). For immunohistochemistry and immunofluorescence, sections were stained overnight at 4 °C with antibodies against Ki67 (Abcam, ab16667,1:300 for IHC and 1:100 for IF), RELMα (Abcam, ab39626, 1:300 for IHC and 1:50 for IF), HIF2α (Abcam, ab199, 1:50), CC10 (Santa Cruz, sc9772, 1:50) and acetyl-tubulin (Sigma-Aldrich, clone 6-11B-1, 1:3000). For immunodetection of BrdU positive cells, the antibody (BD Biosciences, 347580, 1:50) was incubated for 1 h at room temperature. The percentage of Ki67 and BrdU-positive cells in each experimental condition was calculated by counting bronchial epithelial cells in different bronchial airways of each mouse.

### Statistics

Data were expressed as mean ±  standard errors of the mean (SEM). All statistical analyses were performed using GraphPad Prism 5 software. The differences between two groups with similar variances were analysed with a two-tailed Student´s t-test. One-way ANOVA with Tukey’s post-test was performed to compare the variance between multiple groups. A p value of <  0.05 was considered significant: * p <  0.05, ** p <  0.01 and ***p <  0.001.

## Additional Information

**How to cite this article**: Torres-Capelli, M. *et al.* Role Of Hif2α Oxygen Sensing Pathway In Bronchial Epithelial Club Cell Proliferation. *Sci. Rep.* **6**, 25357; doi: 10.1038/srep25357 (2016).

## Supplementary Material

Supplementary Information
