# Supplementary material for: Role Of Hif2α Oxygen Sensing Pathway In Bronchial Epithelial Club Cell Proliferation
Source: Sci Rep. 2016 May 6;6:25357. doi: 10.1038/srep25357 (PMC4858655; doi:10.1038/srep25357)
Supplement: Supplementary Information [file srep25357-s1.pdf]

ROLE OF HIF2α OXYGEN SENSING PATHWAY IN BRONCHIAL EPITHELIAL CLUB CELL PROLIFERATION

Mar Torres-Capelli[1], Glenn Marsboom[2], Qilong Oscar Yang Li[1], Daniel Tello[1], Florinda Melendez Rodriguez[1],Tamara Alonso[3], Francisco Sanchez-Madrid[1], Francisco García Rio[4], Julio Ancochea[3] and Julián Aragonés[1]*.

SUPPLEMENTARY INFORMATION

Supplemental figure 1

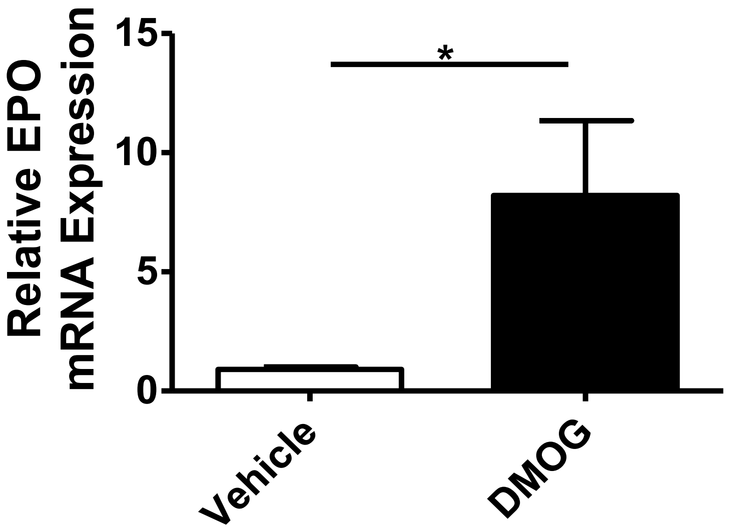

Supplemental Figure 1. Induction of Epo gene expression in the kidney of DMOG-treated mice.

Relative Epo mRNA levels (normalized to those of Hprt) in the kidney of DMOG treated mice (n=3) or vehicle-treated mice (n=2). The differences between

groups with similar variances were analysed with a two-tailed Student´s t-test:$^*p<0.05$.
